# Supplementary figures and images for: Genetic architecture and selective sweeps after polygenic adaptation to distant trait optima
Source: PLoS Genet. 2018 Nov 19;14(11):e1007794. doi: 10.1371/journal.pgen.1007794 (PMC6277123; doi:10.1371/journal.pgen.1007794)

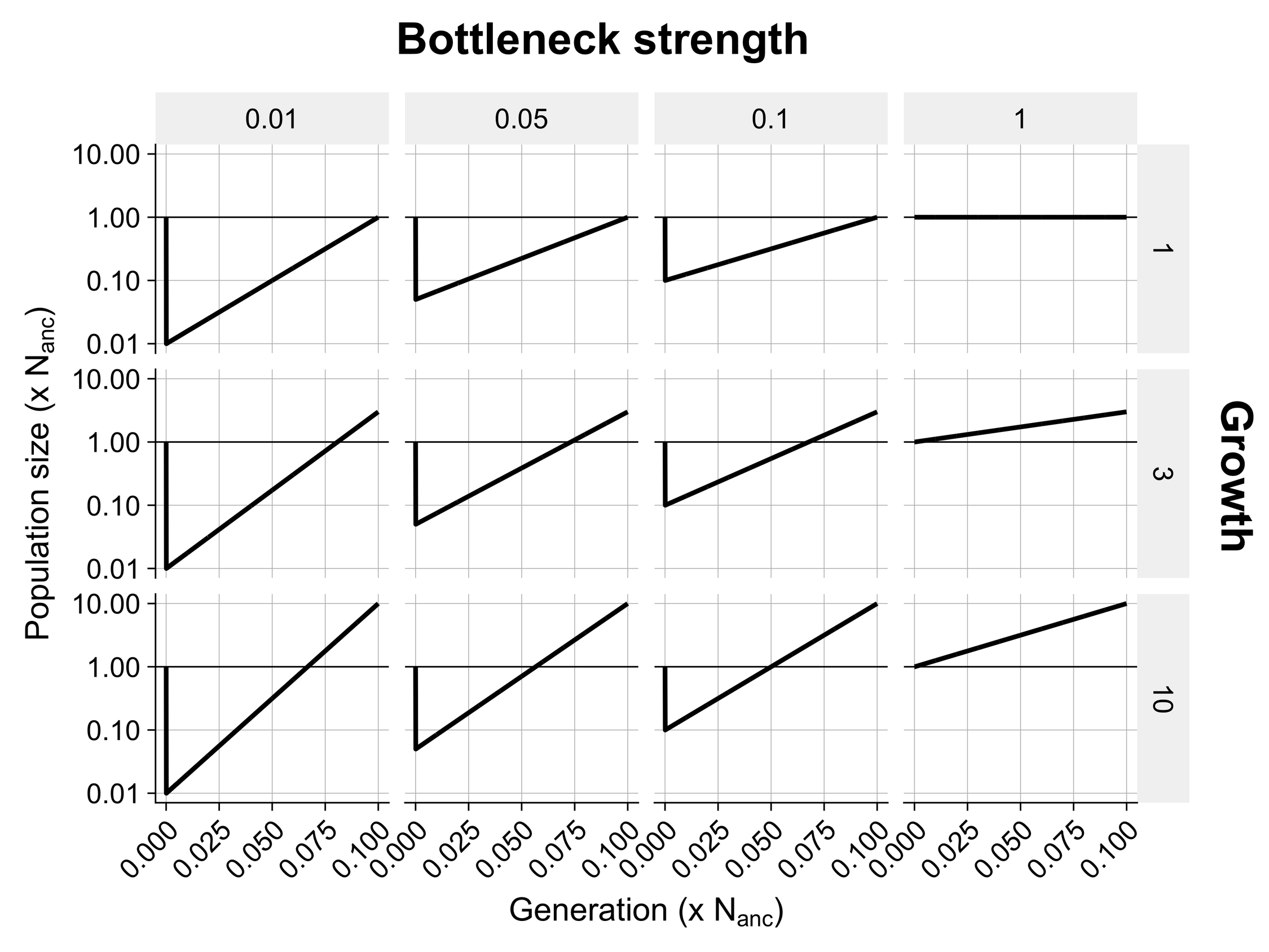

Supplement: S1 Fig — Bottlenecks and growth models. (TIF) [file pgen.1007794.s001.tif]

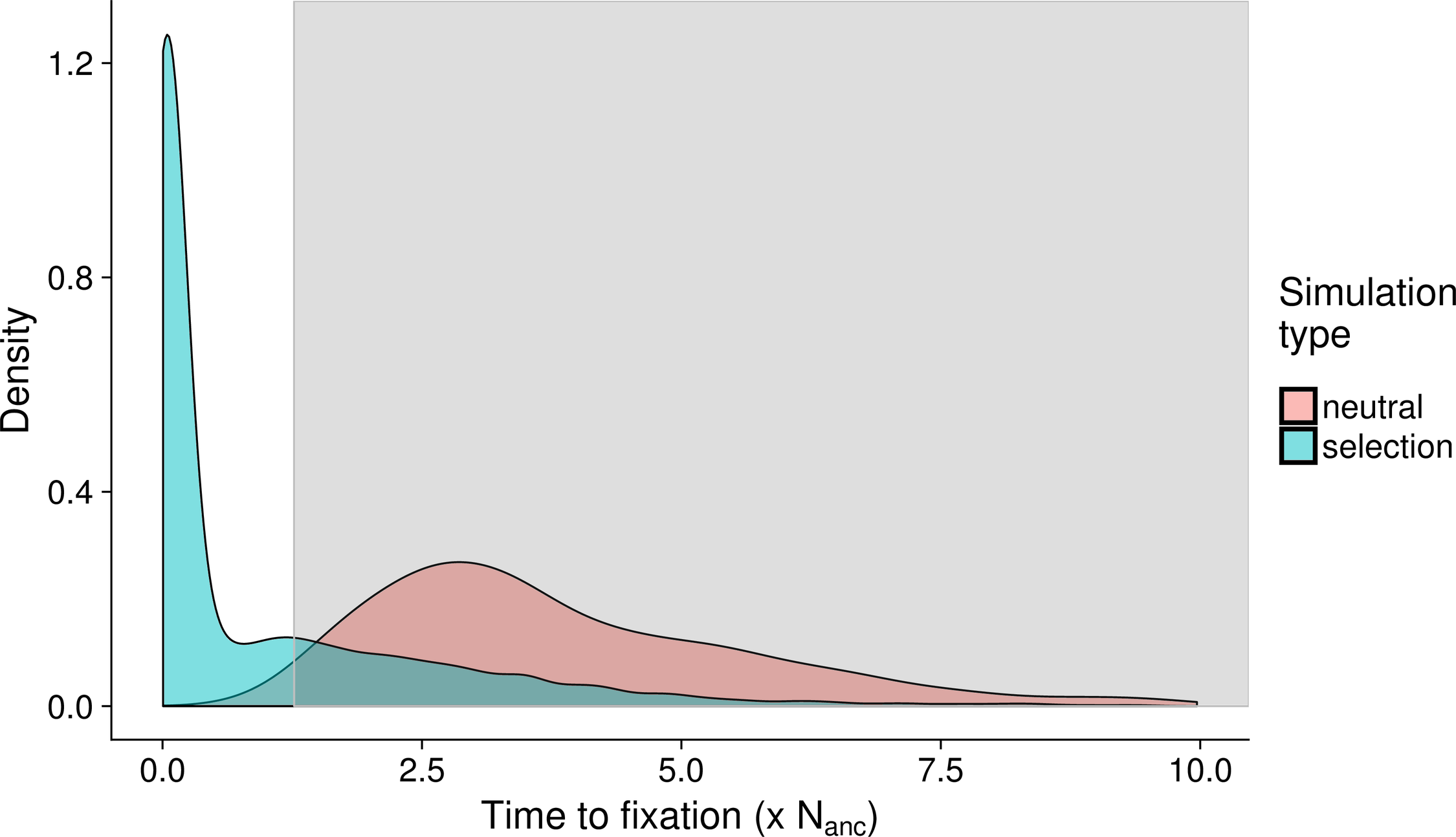

Supplement: S2 Fig — Distribution of fixation times from neutral single locus simulations (red) and forward simulations with selection (green). The grey area denotes the 99% confidence interval of neutral fixation time. Fixations outside the confidence interval are considered selective sweeps. (TIF) [file pgen.1007794.s002.tif]

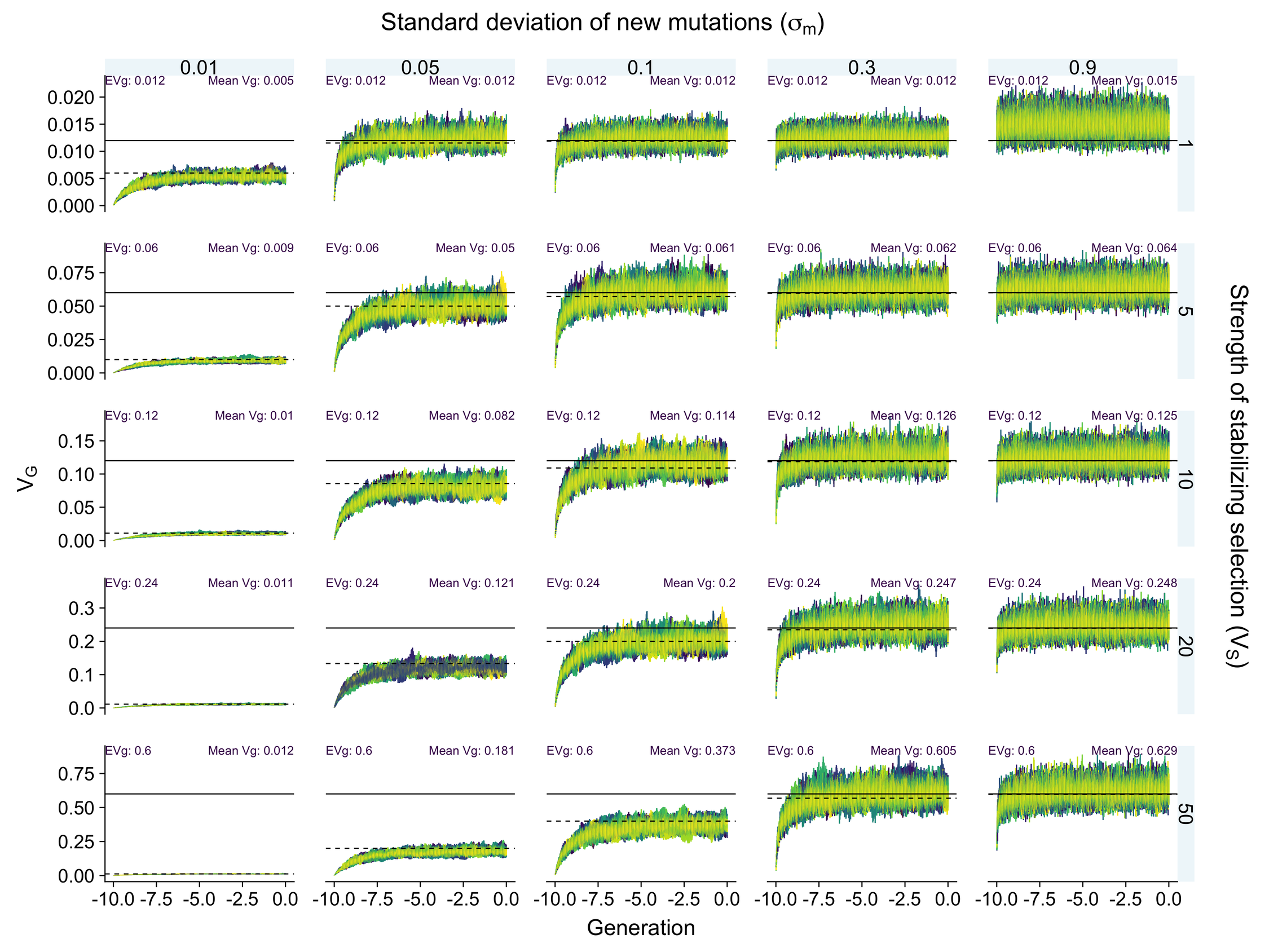

Supplement: S3 Fig — The genetic variance in each generation over 10 Nanc generations for each parameter set. Solid horizontal lines denote the House of Cards approximation of VG [12]. Scenarios with small σm and large VS do not reach the expected VG because mutations are too small to “fill up” the variance volume. However, their equilibrium variance is well approximated by the stochastic House of Cards approximation [27, dashed line]. (TIF) [file pgen.1007794.s003.tif]

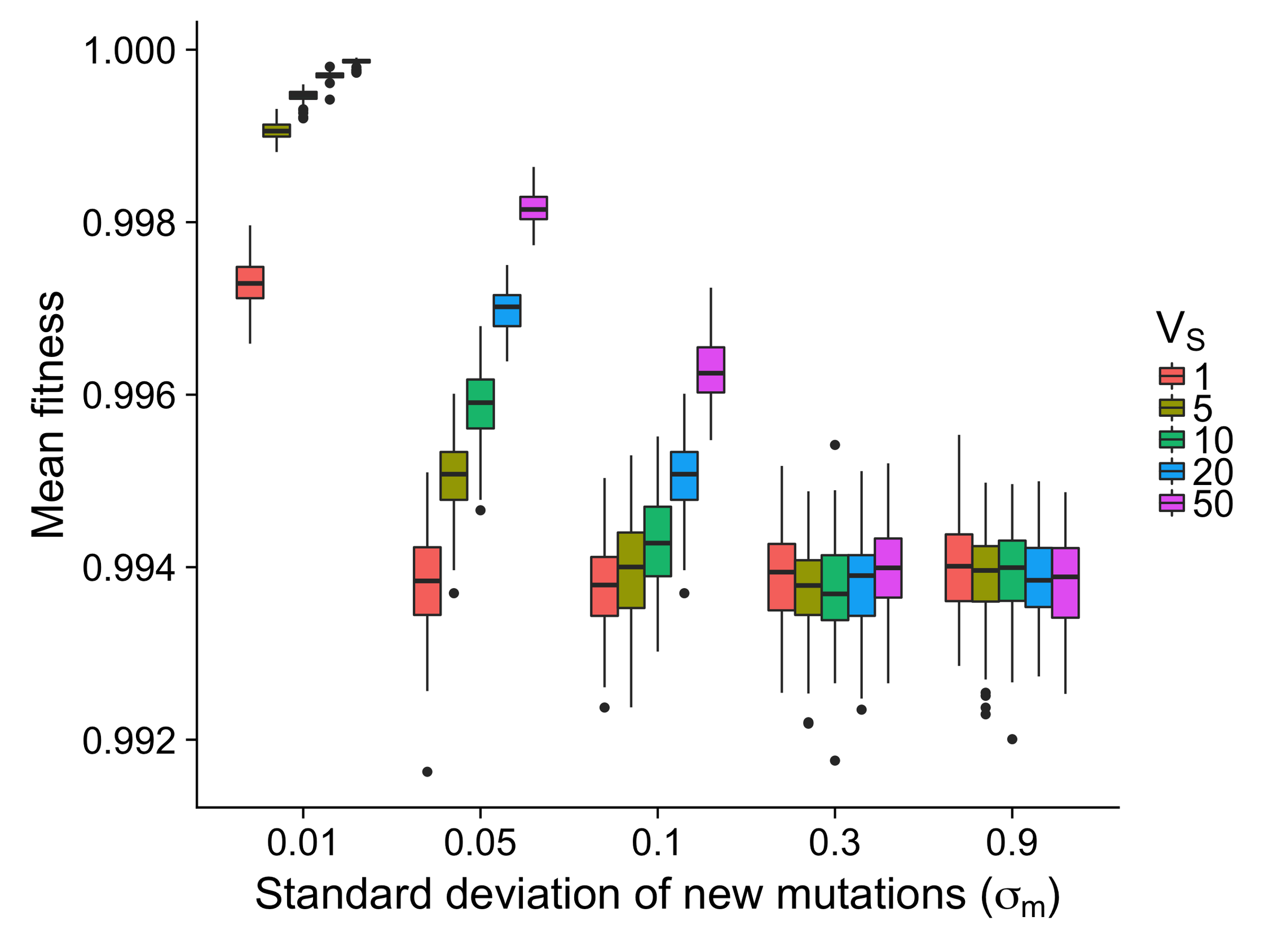

Supplement: S4 Fig — Fitness for each burn-in parameter combination after 10N generations. (TIF) [file pgen.1007794.s004.tif]

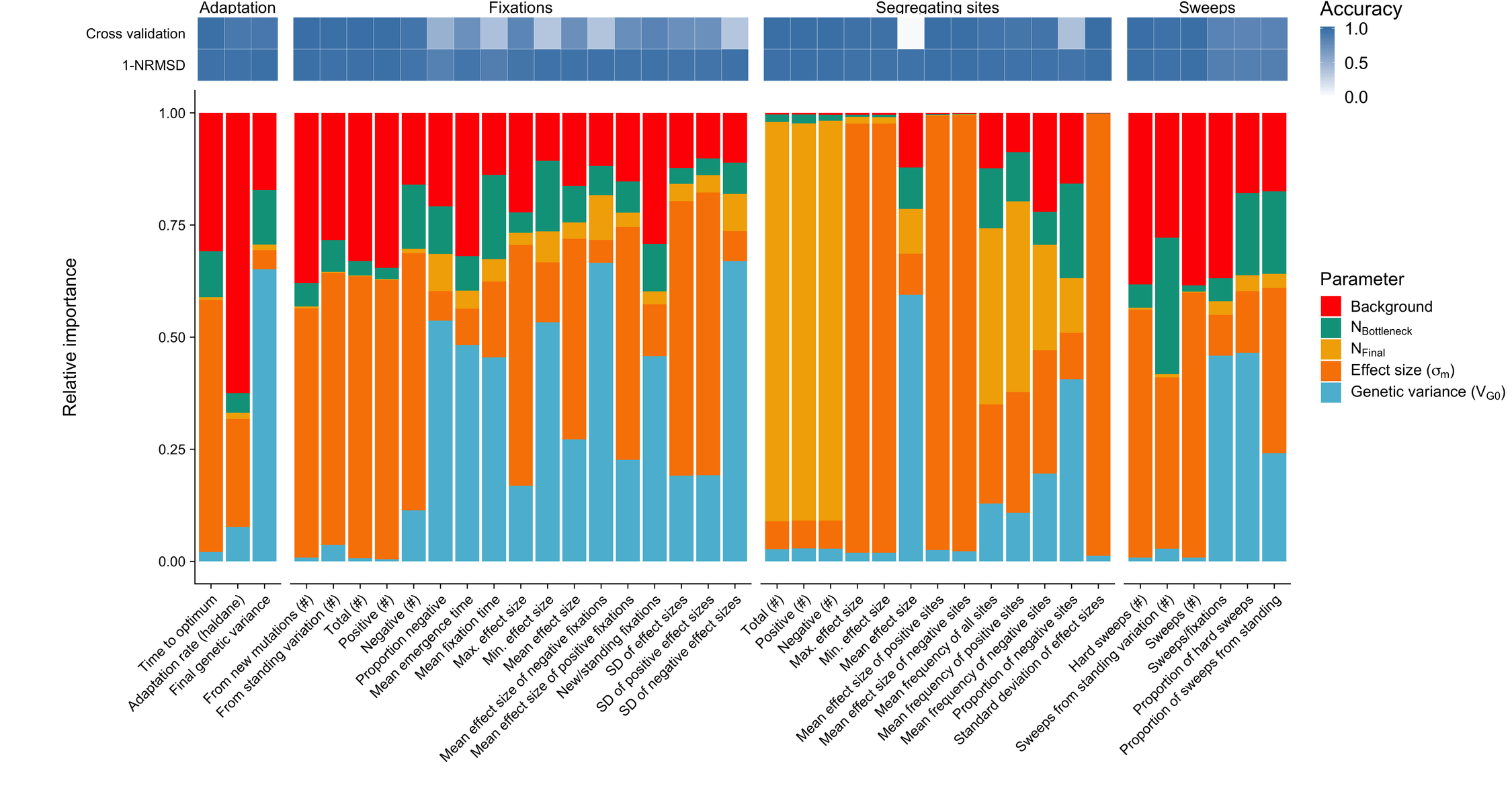

Supplement: S5 Fig — Relative parameter importance inferred by Random Forest machine learning for three parameter categories. 1) Adaptation, trait related parameters describing adaptation speed and potential for future adaptation. 2) Fixations, summary statistics for mutations that were fixed during trait adaptation and 3) segregating sites in the final generation of the simulations. Top panel indicating prediction accuracy as calculated by 10-fold nested cross validation and normalized relative mean squared error. (TIF) [file pgen.1007794.s005.tif]

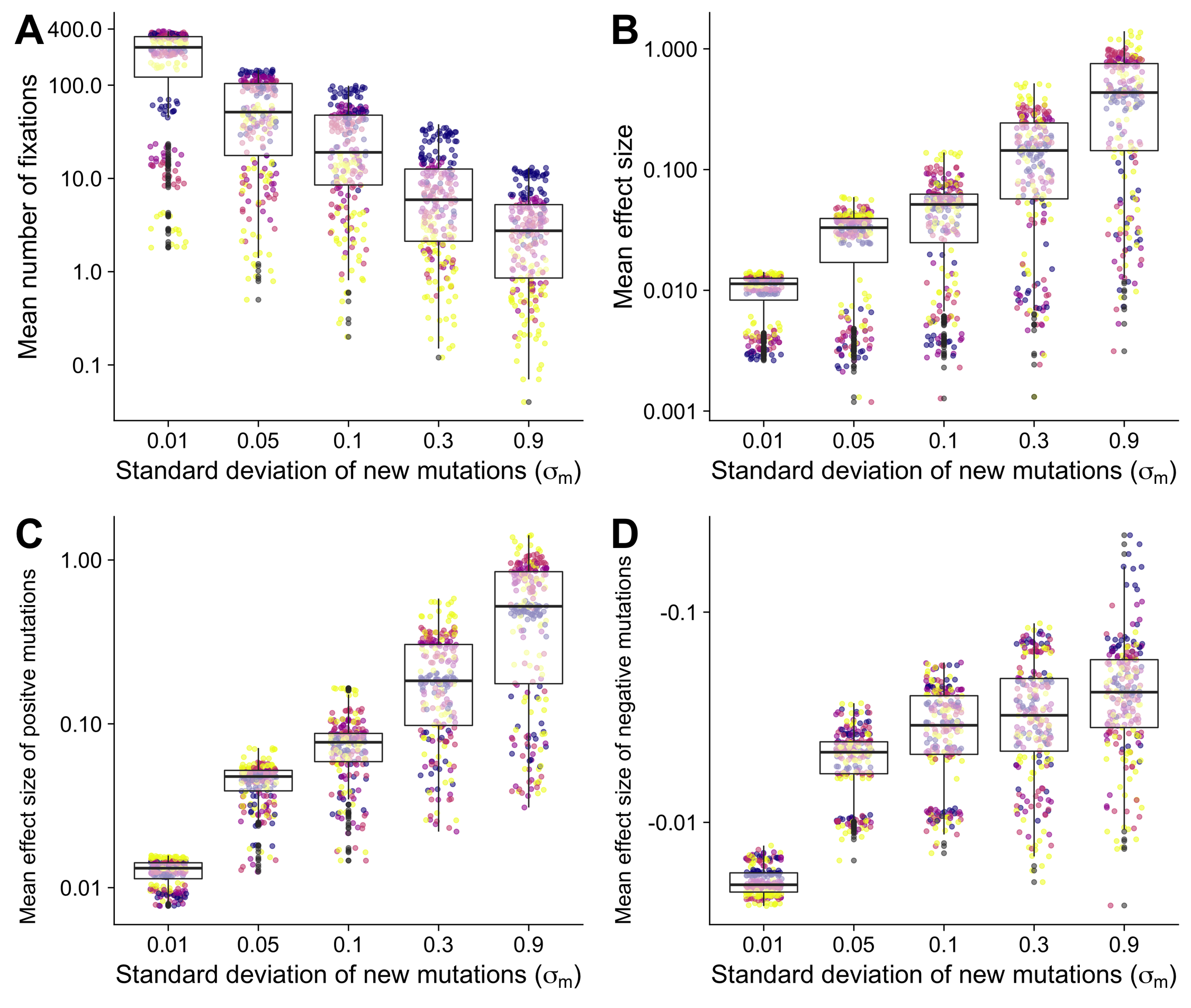

Supplement: S6 Fig — A) Total number of fixations B) Mean effect size of fixations C) Mean effect size of positive fixations D) Mean effect size of negative fixations. (TIF) [file pgen.1007794.s006.tif]

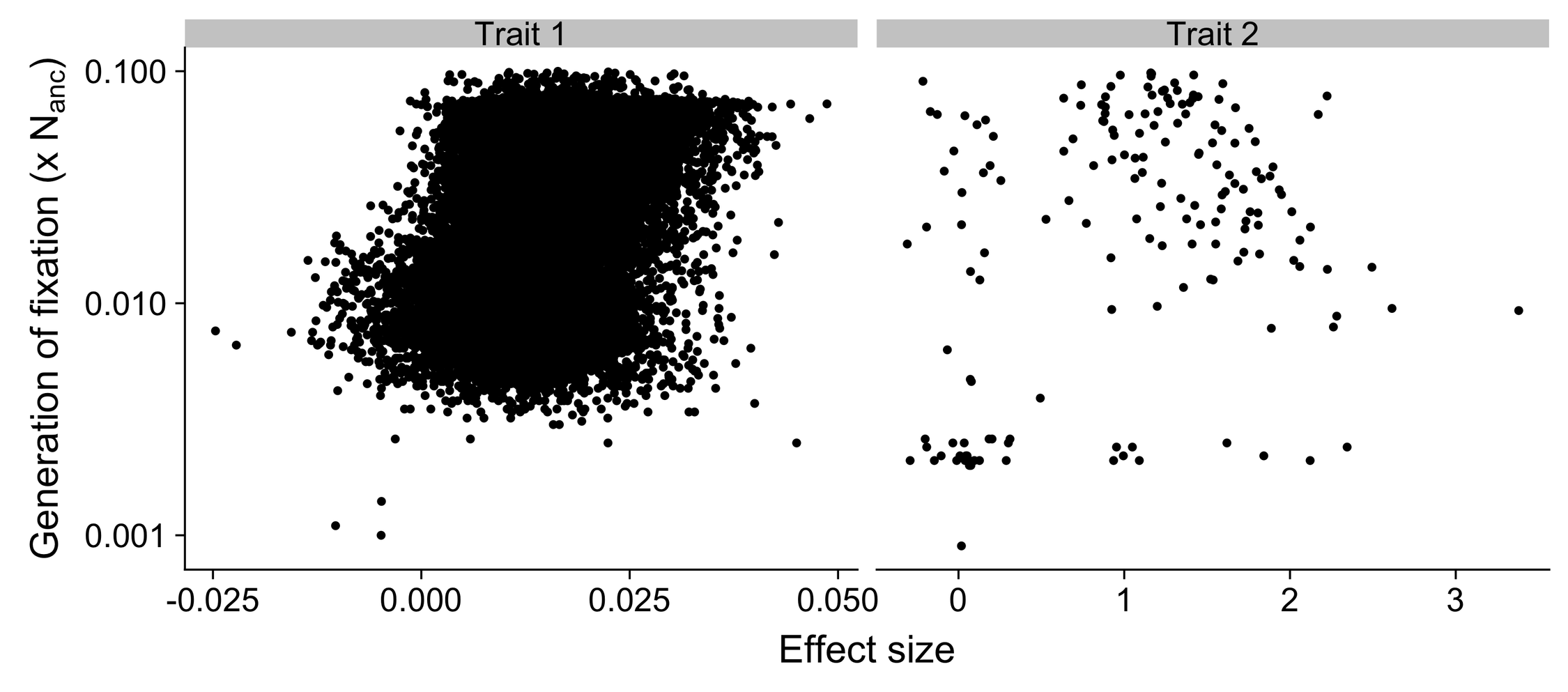

Supplement: S7 Fig — Shown is the relationship between effect size and the generation of fixation for mutations for Trait 1 (left, σm = 0.01 and VS = 1) and Trait 2 (right, σm = 0.9 and VS = 50). (TIF) [file pgen.1007794.s007.tif]

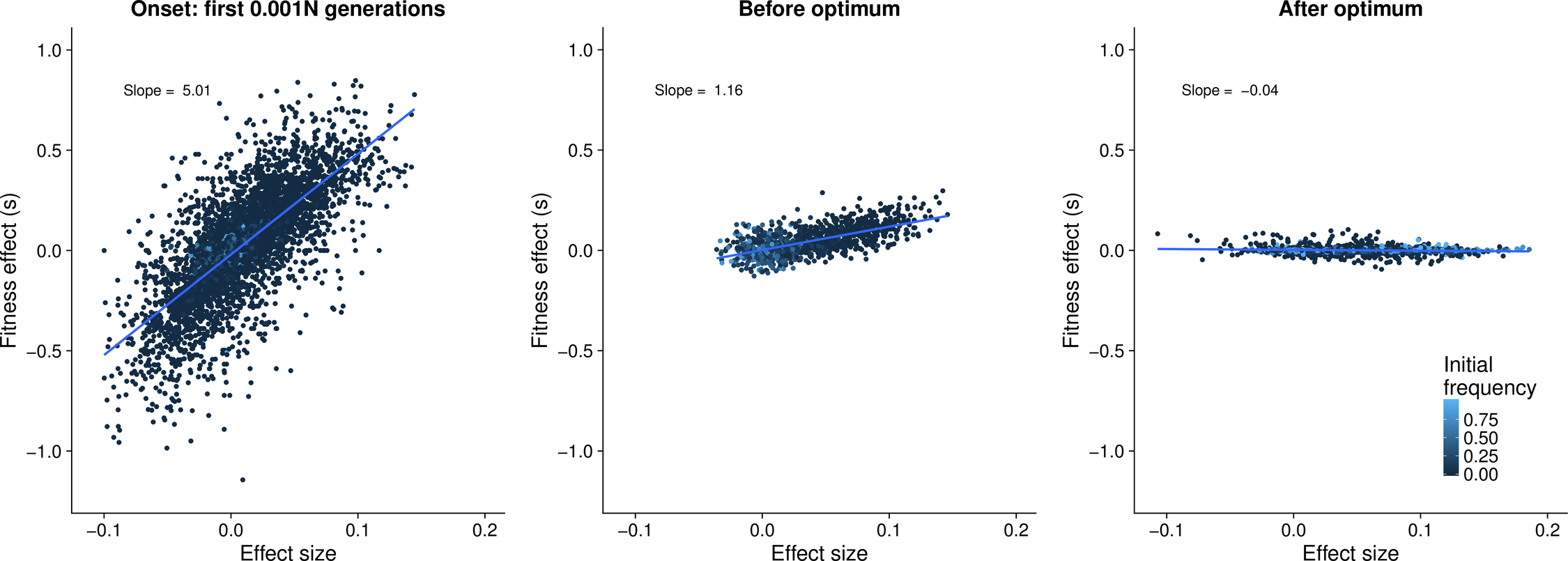

Supplement: S8 Fig — Fitness effects of mutations at the onset of directional selection (0.001–0.012N), before the new optimum is reached (0.001–0.012N) and after the new optimum has been reached (0.012–0.022N). (TIF) [file pgen.1007794.s008.tif]
