# Supplementary material for: Genetic architecture and selective sweeps after polygenic adaptation to distant trait optima
Source: PLoS Genet. 2018 Nov 19;14(11):e1007794. doi: 10.1371/journal.pgen.1007794 (PMC6277123; doi:10.1371/journal.pgen.1007794)
Supplement: S1 Table — (PDF) [file pgen.1007794.s009.pdf]

Table 1: **Predicted summary statistics for feature importance estimation**

Parameter	Description
**Adaptation**	**Trait related parameters**
Time to optimum	Generations until new optimum is reached
Adaptation rate (haldane)	Adaptation rate until new optimum is reached. Calculated as $rate(h) = \frac{\frac{ln(x_2)}{sd_{x_{12}}} - \frac{ln(x_1)}{sd_{x_{12}}}}{t_2 - t_1}$
Final genetic variance	Genetic variance in the final generation
**Fixations**	**Mutations that fix after the optimum shift**
From new mutations (#)	Sum of fixed mutations in the final population that were already segregating before the optimum shift
From standing variation (#)	Sum of fixed mutations in the final population that arose after the optimum shift
Max. effect size	Maximal effect size of all fixations
Mean effect size	Mean effect size of all fixations
Mean effect size of negative fixations	Mean effect size of negative mutations
Mean effect size of positive fixations	Mean effect size of positive mutations
Mean emergence time	Mean generation when a mutation arose that fixed in the last 0.1 N generations
Mean fixation time	Mean generation in which a mutation fixed
Min. effect size	Minimal effect size of all fixations
Negative (#)	Sum of fixed mutations with negative effects in the final population
New/standing fixations	Ratio of mutations from new mutations vs. standing mutations
Proportion negative	Proportion of negative fixations from all mutations
Positive (#)	Sum of fixed mutations with positive effects in the final population
SD of effect sizes	Standard deviation of effect sizes of all fixations
SD of negative effect sizes	Standard deviation of effect sizes of negative fixations
SD of positive effect sizes	Standard deviation of effect sizes of positive fixations
Total (#)	Sum of fixed mutations in the final population
**Sweeps**	**Mutations that fix faster than 99% of neutral fixations**
Hard sweeps (#)	Sum of selective sweeps from new mutations
Proportion of hard sweeps	Porportion of hard selective sweeps of all selective sweeps
Proportion of sweeps from standing	Proportion of selective sweeps from stainding variation of all selection sweeps
Sweeps (#)	Sum of selective sweeps
Sweeps from standing variation (#)	Sum of selective sweeps from mutations that were already segregating before the optimum shift
Sweeps/fixations	Ratio of sweeps vs. fixations
**Segregating sites**	**Mutations that segregate in the final generation**
Max. effect size	Maximal effect size of segregating sites
Mean effect size	Mean effect size of segregating sites
Mean effect size of negative sites	Mean effect size of segregating sites with negative effects
Mean effect size of positive sites	Mean effect size of segregating sites with positive effects
Mean frequency of all sites	Mean allele frequency of segregating sites
Mean frequency of negative sites	Mean allele frequency of segregating sites with negative effects
Mean frequency of positive sites	Mean allele frequency of segregating sites with positive effects
Min. effect size	Minimal effect size of segregating sites
Negative (#)	Sum of segregating sites with negative effect
Positive (#)	Sum of segregating sites with positive effect
Proportion of negative sites	Proportion of segregating sites with negative effect of all segregating sites
Standard deviation of effect sizes	Standard deviation of effect sizes of all segregating sites
Total (#)	Sum segregating sites in the final generation
